# Variable Resistance of RMS to Interferon **γ** Signaling

**DOI:** 10.5402/2012/789152

**Published:** 2012-08-05

**Authors:** Katja Simon-Keller, Katharina Mößinger, Anna-Lena Bohlender, Philipp Ströbel, Alexander Marx

**Affiliations:** Institute of Pathology, University Medical Centre Mannheim, University of Heidelberg, 68135 Mannheim, Germany

## Abstract

*Aims*. Chimeric T cells directed to the **γ**-subunit of the fetal acetylcholine receptor (fAChR) produce large amounts of interferon-**γ** (IFN**γ**) on coculture with fAChR-expressing rhabdomyosarcoma (RMS) cells prior to RMS cell death. The aim of this study was to elucidate whether IFN**γ** blocks proliferation and survival of RMS cells and modulates expression of genes with relevance for cytotoxicity of chimeric T cells. *Methods*. Expression levels of IFN**γ** receptor (IFNGR), AChR, MHCI, MHCII, and CIITA (class II transactivator) by RMS were checked by flow cytometry, qRT-PCR, and western blot. Proliferation and cell survival were investigated by annexin V and propidium iodide staining and MTT (thiazolyl-blue-tetrazolium-bromide) assay. Key phosphorylation and binding sites of IFNGRs were checked by DNA sequencing. *Results*. IFN**γ** treatment blocked proliferation in 3 of 6 RMS cell lines, but reduced survival in only one. IFNGR was expressed at levels comparable to controls and binding sites for JAK and STAT1 were intact. Induction of several target genes (e.g., AChR, MHCI, and MHCII) by IFN**γ** was detected on the RNA level but not protein level. *Conclusions*. IFN**γ** does not significantly contribute to the killing of RMS cells by fAChR directed chimeric T cells. Signalling downstream of the IFNR receptor, including the posttranscriptional level, is impaired in most RMS cell lines.

## 1. Introduction

Interferon gamma (IFN*γ*) plays a crucial role in tumor formation and protects host against growth of spontaneous or transplanted tumors [1, 2]. Besides its immunomodulatory effects, IFN*γ* has an influence on proliferation and induces apoptosis *in vitro* in many primary tumor cells and established tumor cell lines [3–6]. 

IFN*γ* is the only member of the type II interferon family and is mainly produced by activated NK-cells and NKT cells [7], as well as CD4^+^ T-cells and cytotoxic CD8^+^ lymphocytes [8]. The active form of the cytokine is a dimer which binds to a heterodimeric receptor complex that consists of IFNGR1 and IFNGR2 subunits and is associated with two Janus kinase family members, Jak1 and Jak2. Changes in confirmation of receptor subunits after IFN*γ* binding activate Jak1 and Jak2, which in turn phosphorylate IFNGR1 and generate a binding site for recruitment, phosphorylation, and dimerization of signal transducer and activator of transcription 1 (STAT1). After translocation of STAT1 homodimers to the nucleus and binding to GAS (IFN*γ* activated sites) promotor elements, transcription of target genes is initiated [9–11], including MHC class I and II genes with immunomodulation function. Other genes affected by IFN*γ* are the cyclin-dependent kinase inhibitors p21^WAF1/CIP1^ and p27^KIP^ [12], which mediate growth arrest, as well as PI3K, PKC, and different MAPK involved in STAT1 function [13–15]; recently genes such as Bik/Blk/Nbk with an importance for apoptotic pathways have been linked to IFN*γ* response[16]. 

In the current study, we focus on Rhabdomyosarcoma (RMS), the most common form of soft tissue sarcoma, which mainly affects children and adolescents [17, 18]. RMS are subdivided in alveolar RMS (ARMS) and embryonal RMS (ERMS). While overall survival of patients with localized and resectable RMS improved significantly during the last decades, with an overall survival rate of 65%, survival has remained poor in metastatic disease [17, 19, 20]. As a new treatment strategy for RMS, we have used chimeric T cells with a specificity against the fetal acetylcholine receptor (AChR) which is expressed on the surface of RMS [21]. Chimeric T cells are generated by transduction with expression vectors that code for a fully humanized chimeric antigen receptor (CAR) against the AchR*γ* subunit [22]. Binding to target antigen results in strong IFN*γ* secretion by chimeric T cells that exert specific cytotoxicity against RMS cell lines *in vitro* [22, 23]. One of the previous studies suggested that IFN*γ* might significantly contribute to the proapoptotic effects of RMS-directed chimeric T cells [23]. Furthermore, work by Poëa-Guyon et al. revealed that pro-inflammatory cytokines such as IFN*γ* induce overexpression of AChR, that is, the target of chimeric T cells, on the cell surface of RMS-like transformed thymic myoid cells [24]. Therefore, we studied the influence of IFN*γ* on ARMS and ERMS cell lines, showing that most of them are resistant to even high concentrations of IFN*γ* in terms of induction of apoptosis and AChR overexpression.

## 2. Material and Methods

### 2.1. Material

HT29 colon adenocarcinoma cell line was cultured in DMEM, 10% (v/v) FCS. The alveolar RMS cell lines CRL2061, RH30, RH41 (all Pax3-FKHR translocation positive), and FLOH1 (translocation negative) were cultivated in RPMI1640 medium with 10% (v/v) FCS. The embryonal RMS cell lines RD6 and TE671 were maintained in DMEM with 10% (v/v) FCS. 

Recombinant IFN*γ* was purchased from R&D Systems. The demethylation reagent 5′-Aza-2′-deoxycytidine was obtained from Sigma Aldrich (St. Louis, MO, USA). Mouse anti-human AChR antibodies against alpha and gamma subunit were obtained from GeneTex (Irvine, CA, USA); rat anti-human antibodies against alpha and gamma subunit of the AChR were a kind gift from S. Tzartos (Department of Biochemistry, Helenic Pasteur Institute, Athens, Greece); to detect human MHC classII (HLA-DRA), we used a mouse anti-human antibody (clone L243; kind gift from H. Kalbacher; Interfaculty Institute of Biochemistry, University of Thübingen); mouse anti-human IFNGR1 and goat anti-human IFNGR2 antibody were purchased from R&D Systems (Minneapolis, MN, USA). To detect CIITA we used a goat anti-human antibody from Santa Cruz Biotechnology (Santa Cruz, CA, USA), Caspase analysis was done with a mouse anti-human caspase 8 antibody from cell signalling (Danvers, MA, USA).

FITC-conjugated anti-mouse antibody was purchased from R&D and TRI-conjugated antibody from CALTAG Laboratories. The PE-conjugated donkey anti-rat antibody and a FITC-conjugated donkey anti-goat antibody were from Jackson ImmunoResearch. Isotype-matched antibodies or secondary antibodies of irrelevant specificities were used as staining controls (Sigma Aldrich, St. Louis, MO, USA). 

Horse-radish-peroxidases- (HRP-) conjugated antibodies (Santa Cruz) with specific specificity to primary antibodies were used as secondary antibody for western blot analyses. 

### 2.2. Real-Time PCR

Total RNA was extracted from RMS cell lines and biopsy samples using TRIzol reagent (Invitrogen, Carlsbad, CA, USA). Reverse transcription PCR (RT-PCR) was performed using “RevertAid H Minus First Strand cDNA Synthesis Kit” (Fermentas, St. Leon Roth, Germany). PCR amplification was performed by the “Step one plus system” with the following primer oligonucleotides: GAPDH fwd TGCACCACCAACTGCTTAGC; GAPDH rev GGCATGGACTGTGGTCATGAG; AChR*α* fwd AACACACACCACCGCTCAC AChR*α* rev: CTCGATGGCACTTTTCACCT; AChR*γ* fwd: CTGTGCAGGACACCCAGTC; AChR*γ* rev CGGGCCTTTCTCTAGCTTCT; MHCI fwd GAGGCAAGAGTTGTTCCTGC; MHCI rev CTCCCCACCTCCTCACATTA; MHCII fwd TGTAAGGCACATGGAGGTGA; MHCII rev ATAGGGCTGGAAAATGCTGA. The amplification products were detected with “Fast Sybr Green” (both Applied Biosystems, Carlsbad, CA, USA). Data were analysed by using the REST software tool (Qiagen, Hilden, Germany). Expression levels of the target mRNAs were normalized to endogenous GAPDH mRNA.

### 2.3. Sequencing

Sequencing of phosphorylation sites in IFNGR1 was done using ABI BigDye Terminator sequencing kit according to the manufacturer's instructions. The following primers were used for JAK binding site: IFNGR1 JAK fwd: CTGACTGATTGATGGCAGGT, IFNGR1 JAK rev: AGAATTGCAGAGCTGGGAAG and STAT1 phosphorylation site: IFNGR1 STAT fwd: GGAGGTGGTCTGTGAAGAGC and IFNGR1 STAT rev: TCTTTACCGCTATCATCCACAA.

### 2.4. Western Blot Analysis

Cells were washed three times with ice-cold PBS and incubated 30 min in 2% (w/v) SDS, 60 mM Tris pH 6.8, phosphatase, and protease inhibitor cocktail (ProteoBlock, Fermentas, St. Leon-Roth, Germany) on ice. Cellular debris was removed by centrifugation and proteins (20 *μ*g) were separated by 12% (w/v) SDS polyacrylamid electrophoresis, followed by protein transfer to PVDF membranes (GE Healthcare, Fairfield, CT, USA). Membranes were blocked with 5% (w/v) low-fat milk or BSA (PAA, Pasching, Austria) for 30 min, incubated with primary antibody for 2 h at room temperature or over night at 4°C, washed in TBS, 0,05% (w/v) Tween and incubated with the HRP-conjugated secondary antibody. Binding of antibodies was visualized with the “ECL detection reagent” (GE Healthcare) and documented using the Chemi-smart 5100 (PEQLAB, Erlangen, Germany).

### 2.5. Cytotoxicity Assay

To analyze apoptotic effects towards target cells after different incubation periods with IFN*γ*  1 × 10^4^, tumor cells per well were seeded out in 96 well plates and incubated with 1% FCS 24 h before IFN*γ* treatment, followed by addition of 100 ng/mL IFN*γ* and incubation for 0, 24, 48, 72, and 96 h. Cells were then incubated for 4 h with 20 *μ*L MTT (5 mg/mL). MTT salt was solved in 200 *μ*L DMSO and reduction of MTT by viable tumour cells was colorimetrically determined at an adsorbance wavelength of 560 nm and a reference wavelength of 670 nm. The viability of tumour cells was calculated as the mean of three wells containing tumour cells, the background as the mean of three wells containing medium. Survival of nontreated tumour cells (0 h) was set 100%.

For demethylation experiments with 5′aza-2′-deoxycytidine (Sigma Aldrich, St. Louis, MO, USA) cells were pretreated with 10 *μ*M reagent and 1% FCS and incubated for 72 h before treatment with IFN*γ* as described [12].

### 2.6. Flow Cytometry

2 × 10^5^ cells were used per staining. After 3 washings in PBS primary antibodies were incubated for 1 h (4°C) and removed by washing with PBS; secondary antibody was incubated for 20 min at 4°C and removed by washing with PBS; flow cytometry analysis was performed on a BD FACSCalibur flow cytometer.

Detection of apoptotic and necrotic effects after incubation with 100 ng/mL IFN*γ* was monitored using Annexin V and propidium iodide staining. Before IFN*γ* treatment cells were cultivated in 1% FCS for 24 h and incubated with 100 ng/mL IFN*γ* for 0, 24, 48, 72, and 96 h. At the end of the incubation period cells were collected by trypsination, washed three times in PBS, resuspended in 100 *μ*L Annexin V binding buffer, stained with 5 *μ*L Annexin V and 10 *μ*L PI (both Biolegend, San Diego, CA, USA) for 15 min at room temperature, and analyzed by flow cytometry after addition of 400 *μ*L Annexin V binding buffer.

### 2.7. Statistical Analysis

For statistical analysis an unpaired *t*-test was applied using the GraphPad Software (San Diego, CA, USA). 

## 3. Results

### 3.1. RMS Cells Are Highly Resistance against IFN*γ*-Induced Cell Death

As shown before [23], killing of RMS cells following coculture with fAChR-specific chimeric T cells is preceded by the production of large amounts of IFN*γ*. To examine whether IFN*γ* contributes to RMS cell death, we treated various RMS cell lines with 100 ng/mL IFN*γ* and determined survival at different time points. The IFN*γ*-sensitive colon carcinoma cell line HT29 served as positive control [25].

**Figure 1:**
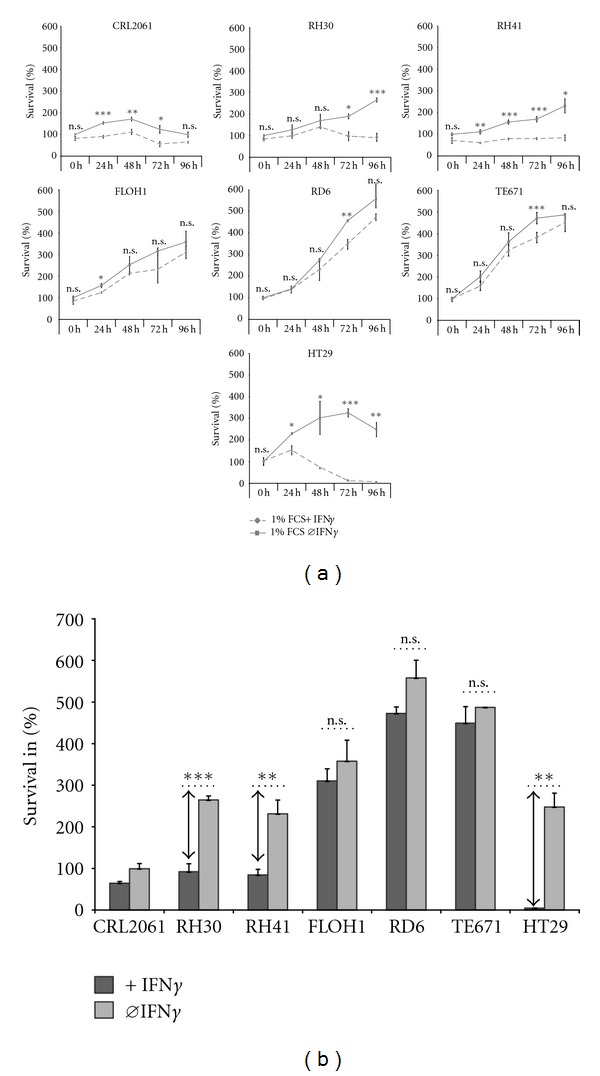
RMS cells are highly resistance against IFN*γ* induced cell death. (a) Survival of RMS cell lines and HT29 control cells after IFN*γ* treatment; cells were incubated for different periods of time with 100 ng/mL IFN*γ* in starvation media with 1% FCS; survival of nontreated tumour cells (0 h) was set 100%. Continuous line reflects cells without IFN*γ* treatment; dashed line correspondent to IFN*γ* treated cells; (b) summarizes effects after 96 h of treatment with (dark grey bars) and without (light grey bars) IFN*γ*. Data represent the mean of triplicates + SEM; one representative experiment out of 3 is shown. Statistical analysis was performed using Student's *t*-test. *P < 0.05; **P < 0.01; ***P < 0.001.

HT29 cells started to undergo apoptosis 24 h after the beginning of IFN*γ* treatment. Their proliferation decreased in parallel, resulting in significantly reduced numbers of viable cells after 48 h (69%) and 72 h (10%) (Figure 1(a)). As opposed to HT29 cells, ERMS cell lines RD6 and TE671 and the translocation negative alveolar ARMS cell line FLOH maintained proliferation and survival during IFN*γ* incubation periods up to 96 h (Figure 1(b)) with only minor effects on cell growth. By contrast, IFN*γ* elicited reduced proliferation and growth arrest without cell death in the translocation-positive ARMS cell lines CRL2061 and RH41 (Figure 1(a)), while only RH30 cells showed a decline in viability after 72 h (Figure 1(a)). 

**Figure 2:**
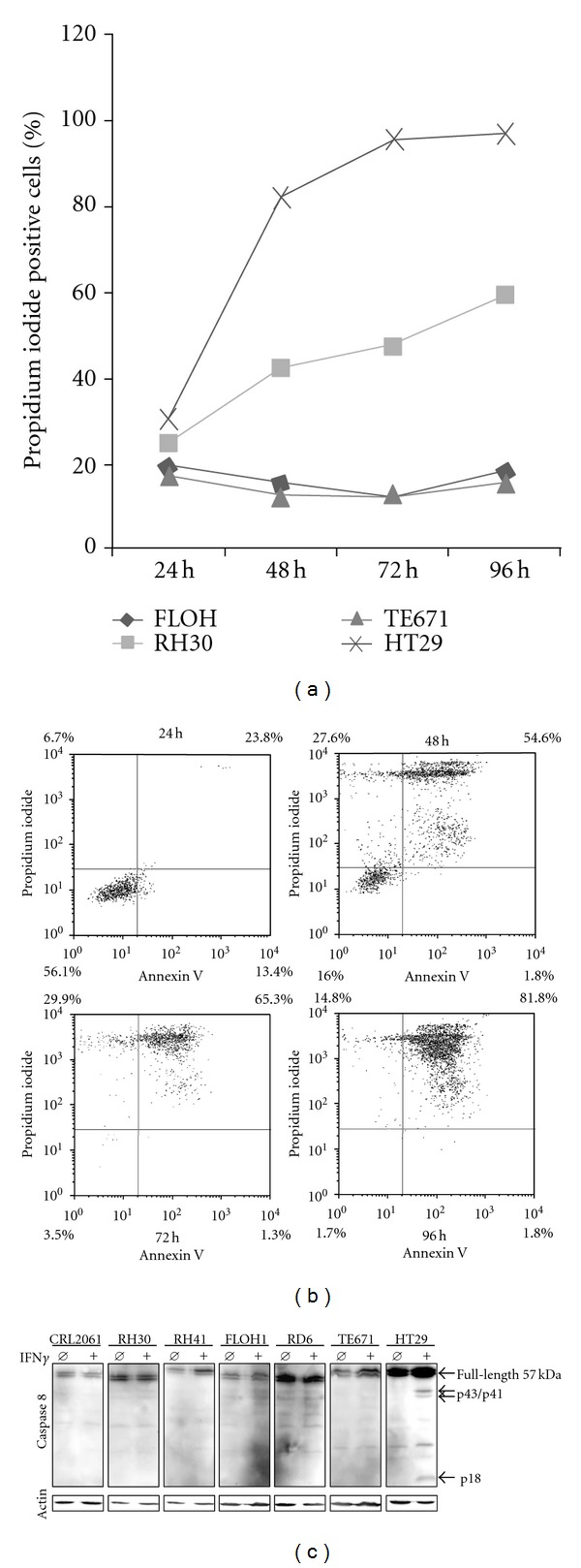
Detection of IFN*γ* induced cell death by flow cytometry and western blot. (a and b) Apoptotic cell detection via flow cytometry of FLOH1, RH30, TE671, and HT29 cell lines after IFN*γ* treatment; cells were incubated for different periods of time with 100 ng/mL IFN*γ* in starvation media with 1% FCS and stained with propidium iodide and annexin V; (a) shows proportion of propidium iodide positive cells after IFN*γ* treatment at different time points; (b) reflects the complete flow cytometry data for HT29 control cell lines; (c) western blot analysis of caspase 8 cleavage 24 h after IFN*γ* treatment; *β*-actin serves as loading control.

Apoptosis was checked in RH30, FLOH1, TE671, and HT29 cells by Annexin V/Propidium iodide (PI) double staining and caspase 8 cleavage assay. Percentage of PI positive cells after 96 h of treatment approached 100% in HT29 cells, 60% in RH30 cells and <20% in the other, IFN*γ*-resistant cell lines (Figures 2(a) and 2(b)). Surprisingly, caspase 8 cleavage after 24 h (Figure 2(c)), 48 h, and 96 h (not shown) was only observed in HT29 cells but not in any RMS cell line tested, including apoptosis-prone RH30 cells (Figure 2(c) and data not shown).

### 3.2. RMS Cells Show Intact IFNRs and STAT1 Phosphorylation *In Vitro*

**Figure 3:**
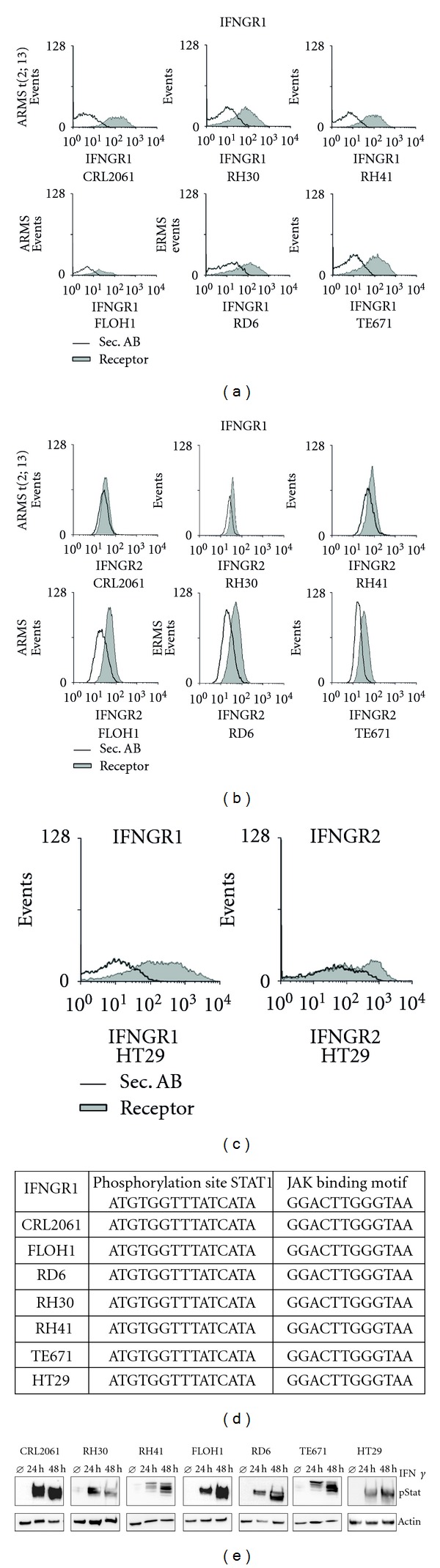
RMS cells show intact IFNRs and STAT1 phosphorylation *in vitro*. (a, b, and c) Flow cytometry data of IFNGR subunit expression in RMS and HT29 control cells lines; filled histograms represent expression levels using specific antibodies; open histograms represent isotype control stainings; (d) mutation analysis of STAT1 phosphorylation site and JAK binding motif in RMS cell lines and HT29 control cells; (e) western blot analysis of p STAT1 induction 24 h and 48 h after IFN*γ* treatment; *β*-actin serves as loading control.

Since IFN*γ* resistance could be due to diminished expression of IFNGR subunits, we next analyzed expression of the IFNGR1 and IFNR2 subunits on RMS cell lines. Apart from CRL2061 cells, that showed barely detectable IFNGR2 expression levels by FACS, both subunits were expressed on the surface of the other RMS cell lines (Figures 3(a) and 3(b)). IFN*γ* treatment (48 h) induced normal [26] decline of IFNGR1 by receptor internalization (not shown) in all tested cell lines. Sequencing of essential phosphorylation sites for JAK binding and STAT1 phosphorylation revealed wild-type sequences (Figure 3(d)). Furthermore, we found that RMS cell lines express high levels of pStat after different incubation periods with IFN*γ* (Figure 3(e)).

### 3.3. IFN*γ* Treatment Does Not Alter Protein Expression of FAchR and MHCII by RMS cells

**Figure 4:**
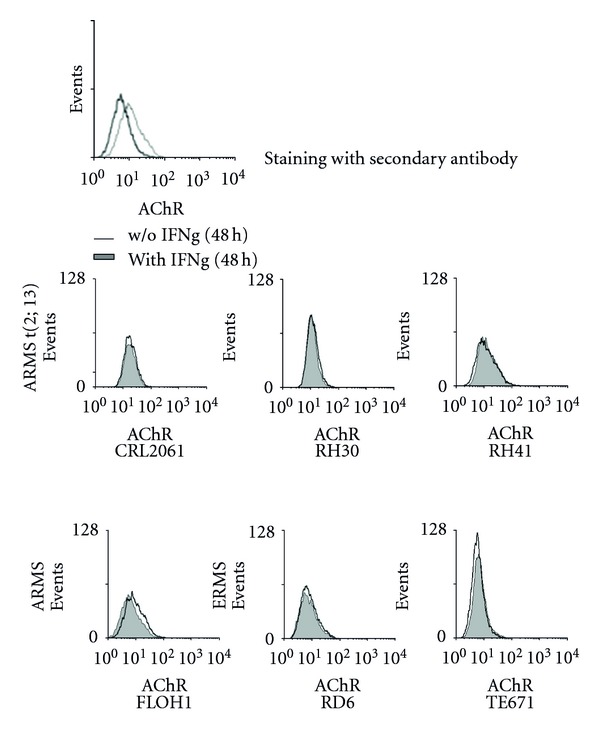
IFN*γ* treatment does not alter protein expression of AChR. Induction of AChR*γ* in RMS cell lines 48 h after IFN*γ* treatment (100 ng/mL); filled histograms represent expression levels after incubation with IFN*γ*; open histograms represent expression levels without IFN*γ* incubation.

**Figure 5:**
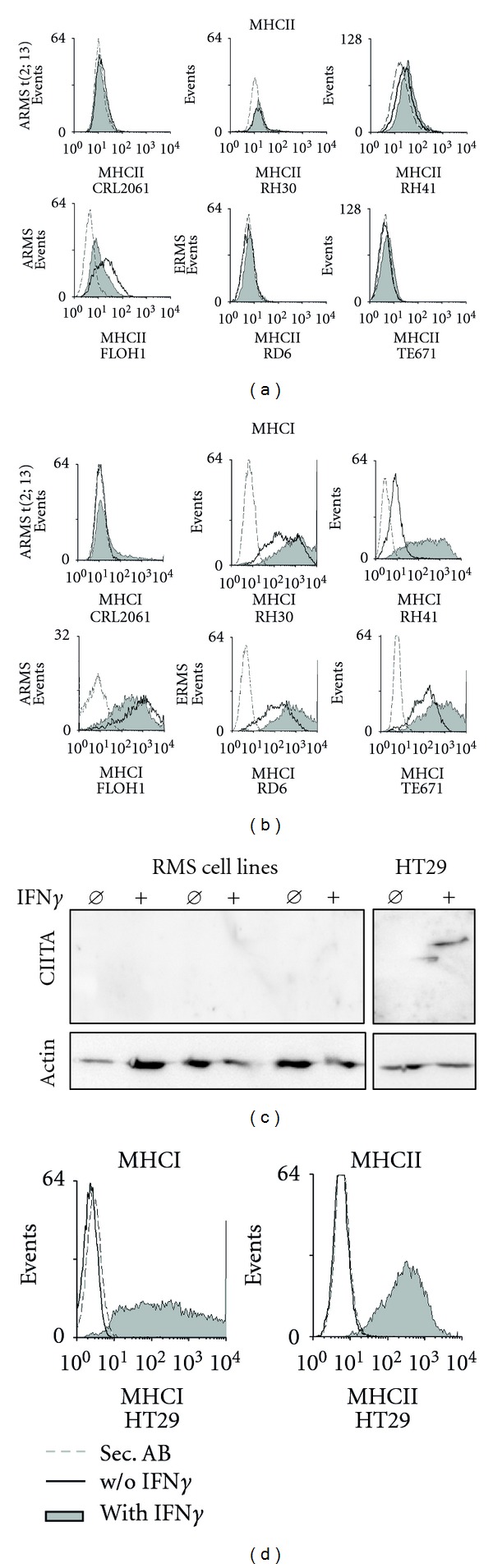
IFN*γ* treatment does not alter protein expression of MHCII by RMS cells. (a, b) Induction of MHCII and MHCI in RMS cell lines and (d) in HT29 control cells 48 h after IFN*γ* treatment (100 ng/mL); grey filled histograms represent expression levels after incubation with IFN*γ*; open histograms represent expression levels without IFN*γ* incubation; broken lines represent the negative control (secondary antibody). In (d), the broken lines overlap with the lines of the open histograms; (c) western Blot Analysis of CIITA induction 48 h after IFN*γ* treatment (100 ng/mL); *β*-actin serves as loading control.

To check whether resistance of most RMS cell lines against IFN*γ*-mediated killing reflects a facet of a broader block of IFN*γ*-driven gene expression, we analyzed AChR and MHC expression on RMS cell lines after incubation with IFN*γ* for up to 72 h. In contrast to a previous report about IFN*γ*-driven AChR induction in RMS-like transformed myoid cells [24], AChR expression on RMS cell was not altered either by IFN*γ* treatment alone (Figure 4) or when combined with TNF*α* (data not shown). As to bona fide IFN*γ* targets, expression of MHC class II and its upstream regulator, CIITA, was not inducible in any RMS cell line (Figures 5(a) and 5(c)), while MHC class I expression was slightly inducible in RH41, RD6, and TE671 but only marginally in CRL2061, RH30, and FLOH1 cells (Figure 5(b)). Of note, IFN*γ*-susceptible, apoptosis-prone HT29 cells exhibited strong induction of MHCI, MHCII (Figure 5(d)), and CIITA (Figure 5(c)) expression on IFN*γ* treatment.

### 3.4. Transcriptional Increase of Target Genes after IFN*γ* Treatment

**Figure 6:**
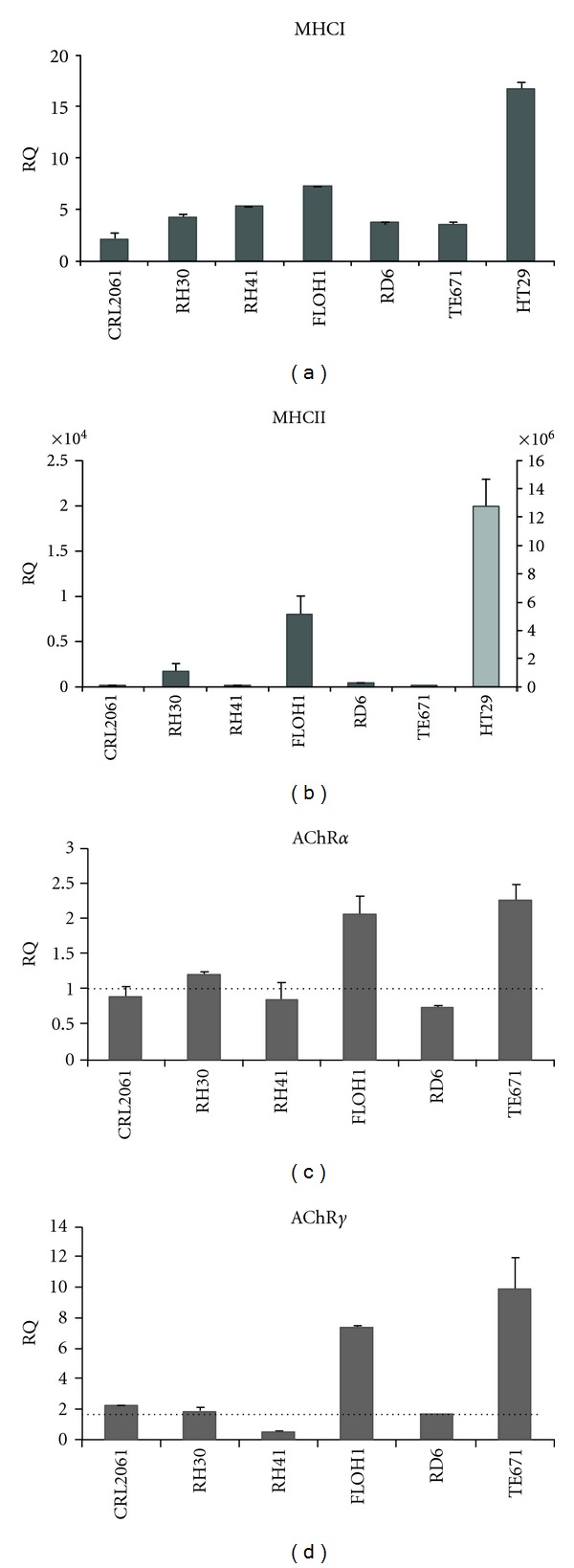
Transcriptional increase of target genes after IFN*γ* treatment. Relative mRNA expression of MHCI (a) and MHCII (b) in RMS cells and HT29 control cells as well as AChR*α* (c) and *γ* (d) in RMS cells after IFN*γ* treatment was determined by qRT-PCR, normalized to GAPDH specific signals and compared to nontreated cells. Expression of each of the respective nontreated cells was arbitrarily set as 1.0. RQ, relative quotient. Data represent the mean ± SEM of one representative experiment out of three. Beware of different scales used for HT29 cells (light grey bar) in (b).

To check whether the block of AChR and MHC protein expression occurs on the transcriptional or posttranscriptional level, we analyzed expression of MHCI, MHCII, and two AChR subunit genes (*α* and *γ*) by qRT-PCR (Figure 6). IFN*γ* increased MHCI (2- to 7-fold) and MHCII (3- to 8.000-fold) mRNA levels in RMS cells. These increases were much lower than in HT29 cells (17-fold for MHCI and 12.700.000-fold for MHCII). Transcripts of AChR subunits were significantly increased only in FLOH1 and TE671 cells, but neither in the other RMS cell lines nor HT29 cells. 

### 3.5. Blockade of IFN*γ* Response Genes in RMS Cannot Be Abrogated by Demethylation

**Figure 7:**
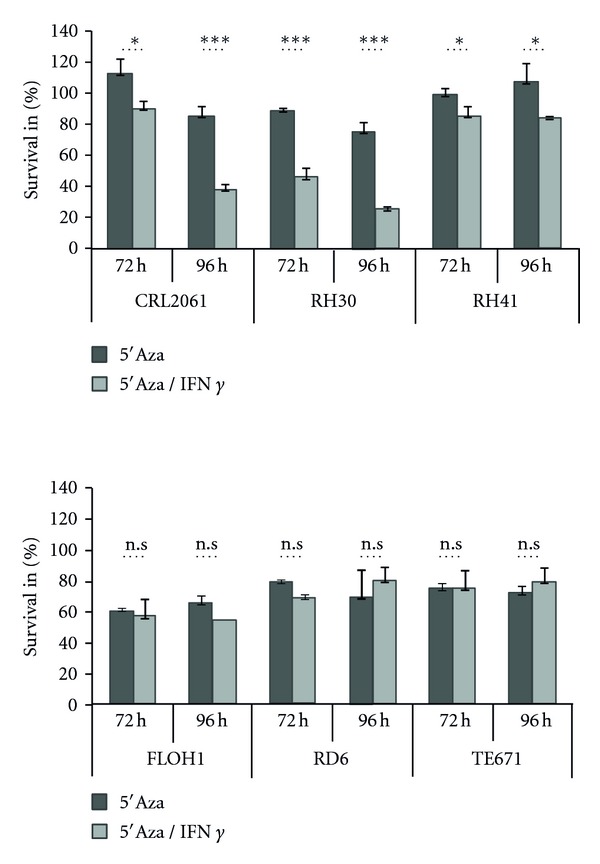
Blockade of IFN*γ* response genes in RMS cannot be abrogate by demethylation. Survival of RMS cell lines after 5′Aza (dark grey bars) and combined treatment of 5′Aza and IFN*γ* (light grey bars); cells were incubated for 72 h with 100 *μ*M 5′aza 2′deoxycytidine (5′Aza) in starvation media with 1% FCS before treatment with IFN*γ* as described; survival of nontreated tumour cells (0 h) was set 100%. *P < 0.05; **P < 0.01; ***P < 0.001.

Chen et al. [27] showed hypermethylation of p21^WAF^ promoter regions in RMS and demethylation with 5′aza 2′deoxycytidine (5′aza) reactivates p21^WAF^ expression. We found similar effects following demethylation that was paralleled by cell cycle arrest in all RMS cell lines. By contrast, demethylation rendered CRL2061, RH30, and RH41 susceptible to IFN*γ*-induced cell death (Figure 7). Furthermore, pretreatment of RMS cell lines with 5′aza had no impact on the defective induction of MHCII or AChR expression by IFN*γ* (data not shown).

## 4. Discussion

In search of novel treatment options for otherwise refractory RMS we generated an immunoreceptor against the RMS-specific fAChR and used chimeric T cells (cTCs) to target RMS cells. However, RMS cell death on coculture with cTCs was rather protracted although cTCs exhibited strong IFN*γ* secretion on antigen recognition [22, 23]. To explain the delayed death response of RMS cells the hypothesis has been put forward that granzyme B-driven apoptotic pathways may be attenuated and that locally secreted IFN*γ* may contribute to RMS cell death [23]. Furthermore, an inductive effect of IFN*γ* on the expression of fAChR, that is, the chimeric T-cell target, has been suggested in RMS-like cells [24]. To address these hypotheses, we here investigated the impact of IFN*γ* on proliferation, apoptosis, and fAChR expression in RMS cell lines. 

Our major finding was that IFN*γ* has antiproliferative effects on CRL2061 and RH41 and apoptotic effects on RH30 while other lines (FLOH1, RD6, and TE671) appeared refractory (Figure 2). However, apoptotic effects even in RH30 cells were smaller than in highly IFN*γ*-sensitive HT29 colon carcinoma cells that served as positive control. In addition experiments with IFN*γ* target genes like MHCI, MHCII, and AChR illustrated a diminished alteration in gene expression after IFN*γ* treatment. Lack of IFNGR2 expression—one of the limiting factors in IFN*γ* signalling [28]—could be excluded (Figure 3). Furthermore, mutations in two essential binding sites in IFNGR1, which are required for receptor function—the JAK binding motive LPKS and Stat1 binding site YDKPH with the essential phosphorylation site Y_440_^30^—were also excluded by sequencing (Figure 3(d)). Indeed, phosphorylation of Stat1 that is necessary for successful IFN*γ* signalling [29, 30], was comparable in RMS cells to phosphorylation in a highly IFN*γ*-sensitive control cell line (Figure 3(e)). 

Since it is known that a broad spectrum of tumor cells lack MHC presentation and show hypermethylation of IFN*γ* target genes such as CIITA [31], we treated RMS cells with the demethylation reagent 5′aza 2′deoxycytidine. Further addition of IFN*γ* resulted in growth arrest and induced cell death in some but not all cell lines (Figure 7). However, induction of MHCII and AChR expression was not achieved. Our results fit in part to those of Chen et al., who described inhibition of cyclin-dependent kinase inhibitor p21^WAF1^ by methylation of SIE-1 promotor elements that resulted in reduced cell cycle control [27] and increased growth. Taken together, hypermethylation of IFN*γ* target genes may be operative in defective cycle control, but may not explain diminished IFN*γ* responses of other target genes. Indeed, the study of Londhe et al. shows that CIITA induction is possible by the combined treatment of RMS cell lines with histone deacetylase (HDAC) inhibitors and demethylation agents, indicating a complex block of accessibility to some promoters in RMS cell lines [32]. However, even this mechanism may not apply to all promoters, considering our finding that some IFN*γ* response genes showed upregulation of transcription that did not translate into protein expression. Therefore, we hypothesize that (a) higher levels of mRNA of IFN*γ* target genes may be required for effective translation, which can be achieved by changes in epigenetic modifications and—not mutually exclusive—(b) there could be a posttranscriptional block, for example, by miRNAs, with influence on IFN*γ*-dependent protein expression [33].

The current findings have therapeutic perspectives. *In vivo*,  defective responsiveness to IFN*γ* is associated with more aggressive tumor behaviour, while IFN*γ*-responsive tumors have a better chance to be kept in check by the immune system [34–36]. Overcoming tumor escape by breaking IFN*γ* resistance in RMS is, therefore, worth to be tested as an adjunct to immunotherapies based on vaccination or adoptive transfer of tumor-reactive cytotoxic effector cells.
